# Subchronic Arsenic Exposure Induces Behavioral Impairments and Hippocampal Damage in Rats

**DOI:** 10.3390/toxics11120970

**Published:** 2023-11-30

**Authors:** Fang Chu, Wenjing Yang, Yang Li, Chunqing Lu, Zhe Jiao, Keming Bu, Zhipeng Liu, Hongna Sun, Dianjun Sun

**Affiliations:** 1 Institute for Endemic Fluorosis Control, Center for Endemic Disease Control, Chinese Center for Disease Control and Prevention, National Health Commission Key Laboratory of Etiology and Epidemiology, Harbin Medical University, Harbin 150081, China; 2020020144@hrbmu.edu.cn (F.C.); 202001064@hrbmu.edu.cn (W.Y.); 2020020141@hrbmu.edu.cn (Y.L.); lcq2020@hrbmu.edu.cn (C.L.); 2021020165@hrbmu.edu.cn (K.B.); 2022020105@hrbmu.edu.cn (Z.L.); 2 Heilongjiang Provincial Key Laboratory of Trace Elements and Human Health & Key Laboratory of Etiology and Epidemiology, Education Bureau of Heilongjiang Province, Harbin Medical University, Harbin 150081, China; jiaozhe@hrbmu.edu.cn; 3 Institute for Kashin Beck Disease Control and Prevention, Chinese Center for Disease Control and Prevention, Harbin Medical University, Harbin 150081, China

**Keywords:** arsenic, behavior, neurological function, hippocampal damage, neurotoxicity

## Abstract

This study investigated the effects of subchronic arsenic exposure on behavior, neurological function, and hippocampal damage in rats. Thirty-two male Wistar rats were divided into four groups and exposed to different concentrations of arsenic in their drinking water for 12 weeks, while weekly water intake and body weight were recorded. Various neurobehavioral tests were conducted, evaluating overall activity levels, exploratory behavior, short-term memory, spatial learning and memory, anxiety-like behavior, and depressive-like states. Arsenic levels in urine, serum, and brain tissue were measured, and histopathological analysis assessed hippocampal damage using hematoxylin and eosin staining. The results demonstrated that arsenic exposure did not significantly affect overall activity or exploratory behavior. However, it impaired short-term memory and spatial learning and memory functions. Arsenic-exposed rats exhibited increased anxiety-like behavior and a depressive-like state. Arsenic levels increased dose-dependently in urine, serum, and brain tissue. The histopathological examinations revealed significant hippocampal damage, including neuronal shrinkage, cell proliferation, irregular structure, disordered arrangement, and vacuolation. These findings emphasize the importance of understanding the impact of arsenic exposure on behavior and brain health, highlighting its potential neurological consequences.

## 1. Introduction

Arsenic, a recognized carcinogen and neurotoxin, poses significant environmental health risks. Chronic exposure to inorganic arsenic, primarily through drinking water, has been associated with various health issues. According to the World Health Organization’s (WHO) 2017 guidelines [1], over 200 million people worldwide have excess arsenic in their drinking water (>10 μg/L) [2]. This issue is particularly prevalent in regions such as Bangladesh [3], India [4], China [5], several countries in South and Latin America [6,7], and even in some developed countries like the UK and the US [8]. Long term exposure to arsenic has been linked to diseases in multiple systems and organs, with neurological damage garnering particular attention.

The neurological consequences of prolonged arsenic exposure include increased learning, memory, and attention dysfunction in adults. Children exposed to arsenic in drinking water have demonstrated slower intellectual development compared to non-exposed children. Furthermore, early childhood exposure to arsenic has negative effects on neurodevelopment that continue to adolescence [9]. Animal studies have indicated that arsenic can accumulate in brain tissue, crossing the serum–brain barrier and leading to hippocampal neuron death and memory impairments.

Animal behavioral experiments play a vital role in neuroscience research, particularly in evaluating animal models of neuropsychiatric disorders [10,11]. Previous studies on arsenic-induced neurotoxicity have focused on specific aspects of damage, such as impaired learning and memory [12], anxiety [13], or specific mechanisms [14]. However, there is a lack of comprehensive investigations into the overall neurological damage caused by arsenic exposure.

The hippocampus is a crucial structure involved in neuronal injury. While its precise function is not fully understood, the hippocampus is believed to be associated with processes such as learning, memory, spatial navigation, and neuropsychological disorders [15]. Different regions within the hippocampus have distinct functions, with the CA1 and CA3 regions linked to memory impairment and the dentate gyrus region associated with the development of neuropsychiatric disorders [16,17].

Examining pathological changes in various hippocampal regions can offer valuable insights into the macroscopic effects of arsenic toxicity, enhancing our understanding of how arsenic exposure impacts the hippocampus. Therefore, this study aimed to investigate the relationship between arsenic neurotoxicity and pathological changes in different hippocampal regions.

## 2. Materials and Methods

### 2.1. Animals and Experimental Design

Thirty-two 3-week-old male Wistar rats of SPF-grade were obtained from Beijing Viton Lihua Laboratory Animal Technology Co., Ltd., Beijing, China (SCXK (Beijing) 2016-0006) under license No. SYXK (Heilong) 2017-008. The SPF-grade Laboratory Animal Centre of Harbin Medical University provided the housing for the experimental animals. The rats were housed in cages with natural diurnal light exposure, ad libitum access to food and water, and maintained at an average room temperature of 22 °C with a relative humidity of 40–60%. The study was approved by the Ethics Committee of Harbin Medical University and adhered to all animal ethical requirements.

After one week of acclimatization and regular feeding, the rats were randomly divided into four groups based on their body weight, using a random number table. The experimental groups were exposed to sodium arsenite (NaAsO_2_, CAS No. 7784-46-5, purchased from Beijing InnoChem Science & Technology Co., Ltd., Beijing, China) solutions at concentrations of 2, 10, and 50 mg/L, while the control group received deionized water. The rats were housed for a duration of 12 weeks. The dose selection for this experiment was determined by combining the information from the literature and the previous experimental findings of the research team [18,19,20]. Throughout the study, the rats had ad libitum access to food and the respective solutions. Water intake was recorded daily per cage for each group, with a 5% attrition rate adjustment, and summarized weekly. Rats were weighed weekly.

### 2.2. Neurobehavioral Assessments

#### 2.2.1. Open Field Test

The open field test (OFT) is a widely used behavioral method to assess exploratory and anxiety-like behavior in rodents. The experiment was conducted following the established methodology with the inclusion of additional observational indicators [21]. The test apparatus consists of a black box measuring 100 cm × 100 cm with a height of 40 cm. The box is divided into 16 equal-sized grids in the video system, with the central area comprising four grids. Rats were placed in the behavioral laboratory for a period of 3 h before the formal experiment to acclimate to the environment. During each experiment, the rats were placed in the central area of the open field, and their movements were recorded using video tracking for a duration of 5 min. To ensure cleanliness and minimize the potential impact of odor cues on behavior, the bottom and four walls of the open chamber were sprayed with a 75% ethanol solution and wiped with clean gauze before introducing each new rat. The measured variables encompassed the following: horizontal score (number of grid crossings), vertical score (number of instances the rats stood on their hind legs), total distance traveled, as well as time spent, distance, and average speed within the central region.

#### 2.2.2. Y Maze

The Y maze test is a widely employed method for evaluating short-term memory capacity in rats. The experiment followed established methodologies [22]. The maze consists of three arms: the starting arm, the novel arm, and the other arm. Each arm measures 50 cm in length, 10 cm in width, and 20 cm in height. The experiment comprised two distinct phases: the training phase and the test phase. During the training phase, a baffle was used to separate the novel arm, allowing the rats to freely explore the starting arm and the other arm for a duration of 5 min. Subsequently, one hour later, the same rat entered the test phase. In this phase, the baffle on the novel arm was removed, enabling the rats to freely explore all three arms for 5 min. A camera was positioned 2 m above the Y maze. The quantified measures included the time spent in and the number of entries into the novel arm during the test phase.

#### 2.2.3. Morris Water Maze

The Morris water maze (MWM) was utilized to assess spatial learning and memory in rats. The experiment followed established methodologies [20]. The experimental apparatus consisted of a black circular pool measuring 120 cm in diameter and 75 cm in height. The pool contains water, with a depth of 50 cm and a controlled temperature ranging from 22 to 24 °C. A platform, 10 cm in diameter, positioned 1 cm above the water surface, served as the escape point for the rats. The pool was divided into four quadrants in the camera system, with the platform located in the center of the third quadrant. The MWM test comprised two components: the place navigation experiment and the spatial probe trial.

The place navigation experiment spanned five days, during which the rats underwent training sessions four times a day. Each session involved placing the rats in the water, facing the inner wall of the pool, from a different quadrant each time. Rats placed in the pool naturally exhibit a tendency to search for an escape platform, enabling the measurement of their escape latency and swimming path. Upon locating the platform, the rats were allowed to remain on it for 10 s to facilitate memory consolidation. In instances where a rat failed to find the platform within 60 s, it was guided to the platform and also allowed to stay for 10 s, with the escape latency recorded as 60 s.

On the sixth day, the spatial probe trial was conducted, during which the platform was removed, and the rats were given 60 s to freely explore the pool. Their swimming trajectories were recorded during this period. The quantitative measurements included the total distance, the time spent in the target quadrant, the distance in the target quadrant, and the number of platform crossings.

#### 2.2.4. Elevated Plus Maze

The elevated plus maze (EPM) is a commonly employed behavioral test utilized to evaluate anxiety-like behaviors in rats. The experiment followed established methodologies [23]. The maze structure is constructed from acrylic material, consisting of two open arms and two enclosed arms with walls, arranged in a cross shape. Each arm measures 50 cm in length and 10 cm in width. The enclosed arms feature a 40 cm high fence with an open upper section, while the central area of the maze comprises a 10 cm by 10 cm open region. The entire apparatus is elevated to a height of 50 cm from the ground. A video monitoring system is employed to record the experimental session, capturing the behavior of the rats.

Prior to commencing the test, the rats are acclimated to the environment by being placed in an open field for a duration of five minutes. Subsequently, an individual rat is positioned in the center of the maze, facing one of the open arms, and its behavior is observed and recorded for a period of five minutes. The quantitative measures include the percentage of entries and the percentage of time spent in the open arm.

#### 2.2.5. Forced Swimming Test

The forced swimming test (FST) is commonly employed to evaluate depression-like behavior in rats. The experiment followed established methodologies [24]. The experimental apparatus consists of a transparent cylindrical pool measuring 20 cm in diameter and 46 cm in height. The water depth is adjusted to 30 cm, ensuring that the rats are unable to touch the tank floor with their hind legs. The water temperature is maintained between 23 °C and 25 °C. The test procedure comprises two phases: a pre-test phase and a test phase. During the pre-test phase, the rats are placed in the tank for a duration of 15 min to allow them to acclimate to the environment. Subsequently, after a 24 h interval, the test phase is conducted. The rats are reintroduced to the tank for a period of six minutes, and their activity during the final five minutes is recorded using cameras. Immobility time is recorded as the duration when the rat shows no active movements (e.g., swimming, climbing and struggling), except for those required to keep its head above water.

### 2.3. Execution of Experimental Animals and Sample Collection

The rats were euthanized by administering an intraperitoneal injection of 10% chloral hydrate combined with analgesics to induce unconsciousness. Subsequently, the brain tissue was promptly isolated. The brain tissue was then divided into two halves: the left hemisphere was fixed in paraformaldehyde, while the other half was rapidly frozen in sterile freezing tubes using liquid nitrogen. The frozen samples were subsequently transferred to a storage temperature of −80 °C.

### 2.4. Determination of Arsenic Content

The total arsenic content in urine, serum, and brain tissues of rats was quantified using atomic fluorescence spectrometry, following the most recent Chinese national standard method (GB/T 5750.6-2006) [25,26]. The samples underwent pretreatment with concentrated nitric acid and perchlorate. Prior to analysis, the digested samples were supplemented with 1 mL of a 100 g/L thiourea ascorbic acid solution and 1 mL of concentrated hydrochloric acid, and then diluted to a final volume of 10 mL. A blank sample was prepared using the same procedure. The standard solution was prepared using a reference standard. The total arsenic content was measured using an atomic fluorescence spectrometer. Within the range of the standard curve, the fluorescence intensity exhibited a linear relationship with the concentration of arsenic ions, enabling the calculation of the arsenic content in the samples.

### 2.5. Histopathological Sectioning of Brain Tissue with Hematoxylin–Eosin Staining

The brain tissue, which had been fixed with paraformaldehyde, underwent a series of procedures including washing, dehydration, transparency, wax embedding, and paraffin embedding. Using a slicing mechanism, the embedded sample was cut into 5-micron slices and placed onto slides. The slides were then subjected to sequential staining with hematoxylin–eosin (HE) dye. After sealing the samples with gum, the sections were examined under a microscope for observation.

### 2.6. Main Reagents

The main reagents were as follows: sodium arsenite (Harbin Medical University, Harbin, China), hematoxylin stain (Biosharp, Hefei, China), eosin stain (Biosharp, Hefei, China), toluene (Tianjin Bodi Chemicals Co., Ltd., Tianjin, China), ethanol anhydrous (Tianjin Fuyu Fine Chemicals Co., Ltd., Tianjin, China), paraformaldehyde (Tianjin Bodi Chemical Co., Ltd., Tianjin, China), concentrated nitric acid (Chengdu Sitiande Biological Company, Chengdu, China), perchloric acid (Shanghai Chemical Reagent No. 2 Factory, Shanghai, China), thiourea (Tianjin Comio Chemical Reagent Co., Ltd., Tianjin, China), ascorbic acid (Tianjin Comio Chemical Reagent Co., Ltd., Tianjin, China), potassium borohydride (Shanghai Chemical Reagent No. 2 Factory, Shanghai, China), and arsenic standard solution (CDC Nutrition Institute, Beijing, China).

### 2.7. Main Instruments

The main instruments used are as follows: a SMART data recording and tracking system (Panlab, Spain), behavioral experimental device (Jiangsu Saionce Biotechnology Co., Ltd., Jiangsu, China), biological tissue dehydrator (Hubei Taiwei Medical Technology Co., Ltd., Hubei, China), biological tissue embedding instrument (Hubei Taiwei Medical Technology Co., Ltd., Hubei, China), optical microscope (Olympus Optical Industries, Japan), thermostatic digestion instrument (Qiqihar Precision Instrument Factory, Qiqihar, China), and an atomic fluorescence spectrometer (Beijing Jitian Instruments Co., Ltd., Beijing, China).

### 2.8. Statistical Analysis

Statistical analysis was performed using SPSS 22.0 (International Business Machines Corporation, New York, NY, USA). The data were presented as mean ± standard deviation, and differences between groups were analyzed using one-way ANOVAs. Pairwise comparisons were conducted, using the Least Significance Difference method (LSD) or Dunnett-T3 test. The *p* value less than 0.05 was considered as significant.

## 3. Results

### 3.1. Effects of Subchronic Arsenic Exposure in Drinking Water on Body Weight, Water Intake, and Arsenic Intake in Rats

**Figure 1:**
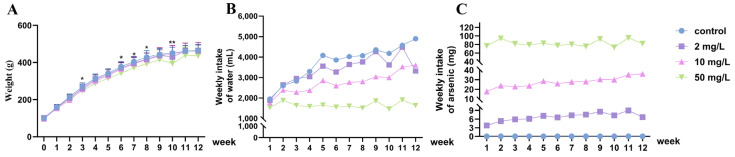
Records of body weight, water intake, and arsenic intake in rats. (**A**) Body weight for 12 weeks of continuous arsenic exposure (g). Values are the mean ± SD. * *p* < 0.05, ** *p* < 0.01, compared with control group, *n* = 8. (**B**) The weekly water intake of the rats in each group (mL). (**C**) The weekly intake of arsenic by the rats in each group (mg).

During the arsenic exposure period, the rats showed normal growth without any significant adverse effects. Starting from the third week, the rats exposed to a high dose of arsenic experienced a noticeable decrease in body weight gain. Additionally, compared to the control group, the rats in the high-dose arsenic-exposed group exhibited significantly lower body weights during weeks 3, 6 to 8, and 10 (*p* < 0.05, Figure 1A). There was a gradual increase in water intake in the control group, while the increase in water intake slowed down with the increasing arsenic concentration. The 50 mg/L group maintained an average water intake of approximately 1700 mL per week (Figure 1B). Despite variations in water intake, the weekly arsenic intake still demonstrated a dose-dependent relationship with the arsenic exposure (Figure 1C).

### 3.2. Effects of Subchronic Arsenic Exposure in Drinking Water on Behavioral Experiments in Rats

#### 3.2.1. Exploratory Capacity

**Figure 2:**
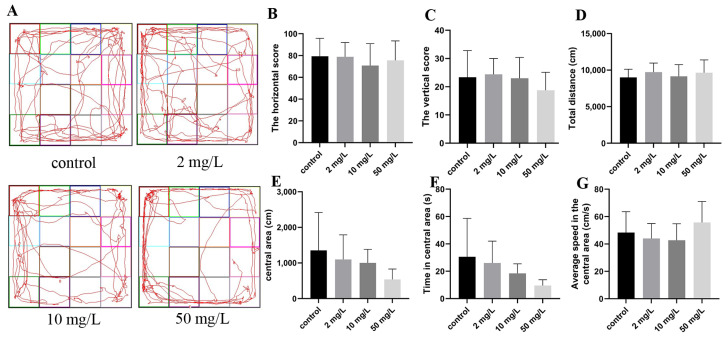
Exploratory capacity was assessed by open field test. (**A**) Representative trajectory diagrams of the open field experiment. (**B**) The horizontal score. (**C**) The vertical score. (**D**) Total distance. (**E**) The distance in the central area. (**F**) Time in the central area. (**G**) Average speed in the central area. Values are the mean ± SD, *n* = 8.

The representative trajectory diagrams the motion trajectory of the rats during the experiment (Figure 2A). The results indicated no significant differences in the horizontal and vertical scores, nor in the total distance traveled by rats across the four groups (Figure 2B–D). The distance and duration of activity in the central area showed a decrease with an increasing arsenic dose, but the observed differences were not statistically significant (Figure 2E,F). Similarly, no significant statistical differences were observed in the speed of the rats in the central region (Figure 2G). These findings suggested that exposure to arsenic did not exert a significant impact on the exploratory behavior or overall activity level of the rats.

#### 3.2.2. Short-Term Memory Capability

**Figure 3:**
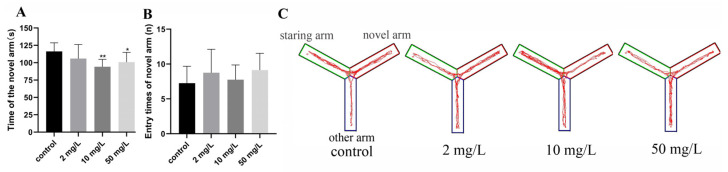
Short-term memory ability was assessed by Y maze. (**A**) Time in the novel arm. (**B**) Entry times of the novel arm. (**C**) Representative trajectory diagrams of Y maze. Values are the mean ± SD. * *p* < 0.05, ** *p* < 0.01, compared with control group, *n* = 8.

In the Y maze task, rats are introduced to a novel environment and form short-term memory to guide their behavior by recalling the arm they have previously entered and actively avoiding revisiting the same arm. The activity time in the novel arm for rats in the medium- and high-dose arsenic-exposed groups were 94.15 ± 10.68 s and 100.80 ± 14.08 s, respectively, which exhibited a significant decrease compared to the control group (116.34 ± 12.29 s) (*p* < 0.01 or *p* < 0.05, Figure 3A). There was no significant difference in the frequency of entering the new arm among all groups (Figure 3B). The trajectory diagram shows the representative paths taken, with the red arm denoting the novel arm. As depicted in the diagram, rats in the control group exhibited a greater inclination to explore the new arm, while the arsenic-exposed group displayed reduced activity in the novel arm (Figure 3C). These findings suggest that arsenic exposure may impair the rats’ short-term memory capability.

#### 3.2.3. Spatial Learning and Memory

**Figure 4:**
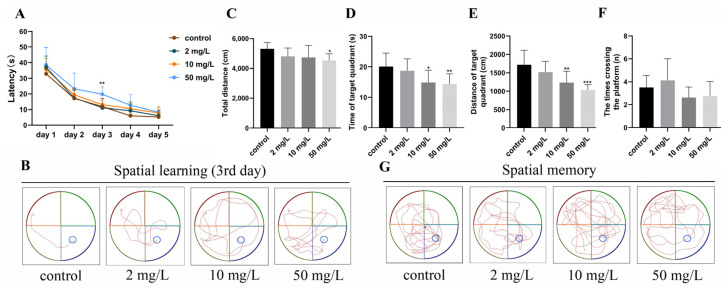
Learning and memory ability were assessed by Morris water maze. (**A**) Latency of rats to find the platform. (**B**) Representative trajectory diagrams of the third day of training. (**C**) Total distance. (**D**) Time of target quadrant. (**E**) Distance of the target quadrant. (**F**) The times crossing the platform. (**G**) Representative trajectory diagrams of space exploration experiment. Values are the mean ± SD. * *p* < 0.05, ** *p* < 0.01, *** *p* < 0.001, compared with control group, *n* = 8.

During the place navigation experiment, the rats acquired spatial memory through the training sessions, enabling them to locate the hidden platform more efficiently. The findings revealed a significant increase in the latency period on day 3 in the high-dose arsenic-exposed group compared to the control group (*p* < 0.05) (Figure 4A). The representative trajectory further illustrated that rats in the control group promptly located the platform upon entering the water, whereas the rats in arsenic-exposed groups took a longer time (Figure 4B).

During the spatial probe trial, the total distance covered by rats in the high-dose arsenic-exposed group was significantly lower than that of the control group (5316.68 ± 427.88 cm vs. 4534.75 ± 453.49 cm, *p* < 0.05), suggesting a potential decline in motivation to seek the platform due to arsenic exposure (Figure 4C). Additionally, the residence time of rats in the target quadrant was significantly decreased in the medium- and high-dose arsenic-exposed groups compared to the control group (14.35 ± 4.05 s and 14.35 ± 3.38 s vs. 20.06 ± 4.44 s, *p* < 0.05 or *p* < 0.01) (Figure 4D). Similar differences were observed in terms of motion distance (Figure 4E). However, there were no significant differences observed in the number of platform crossings among the groups (Figure 4F). The representative trajectory diagram also indicated that rats in the control group explored the areas where the target platform was located more frequently, while those in the arsenic-exposed visited this area significantly less (Figure 4G). Collectively, these findings suggest that arsenic exposure potentially had detrimental effects on spatial learning and memory in rats.

#### 3.2.4. Anxiety State

**Figure 5:**
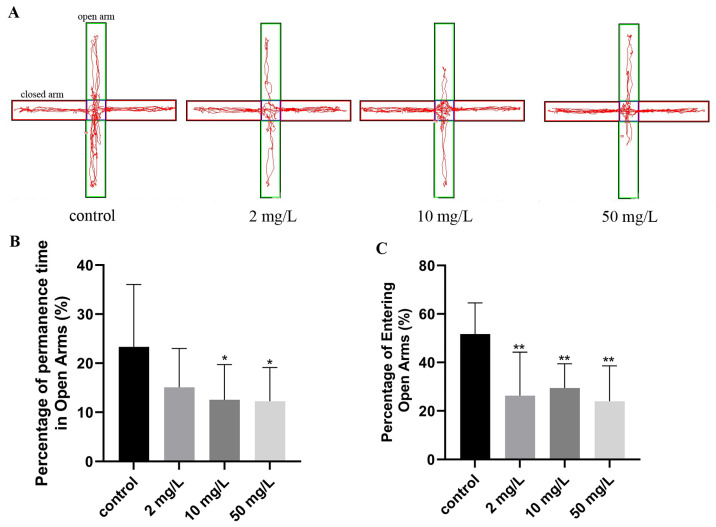
Anxiety state was assessed by the elevated cross maze. (**A**) Representative trajectory diagrams of the elevated plus maze test. (**B**) Percentage of permanence time in open arms. (**C**) Percentage of entering open arms. Values are the mean ± SD. * *p* < 0.05, ** *p* < 0.01, compared with control group, *n* = 8.

The elevated cross maze consisted of open arms represented by green lines and closed arms represented by red lines, as depicted in Figure 5A. Rats in the control group exhibited the longest duration of time spent in the open arm and explored the furthest positions. Conversely, the groups exposed to arsenic demonstrated reduced exploration of the open arm and a shortened exploration distance. The rats’ activity in the open arm showed an inverse correlation with their anxiety tendency. The percentage of residence time in the open arm was significantly lower in the medium- and high-dose arsenic-exposed groups (12.57 ± 7.15%, 12.25 ± 6.86%) compared to the control group (23.33 ± 12.74%) (*p* < 0.05, Figure 5B). Similarly, the percentage of entries into the open arm in the low-, middle-, and high-dose arsenic-exposed groups was 26.28 ± 17.90%, 29.45 ± 10.00%, and 23.96 ± 14.62%, respectively, all significantly lower than the control group (51.73 ± 12.81%) (*p* < 0.01, Figure 5C). These findings support the induction of anxiety in rats following arsenic exposure.

#### 3.2.5. Depression-like State

**Figure 6:**
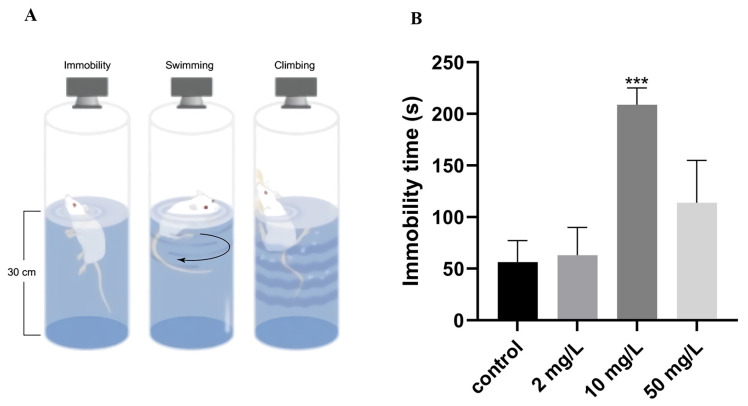
Depression-like state was assessed by forced swimming test. (**A**) Schematic diagram. (**B**) Immobility time. Values are the mean ± SD. *** *p* < 0.001, compared with control group, *n* = 8.

We utilized the forced swimming test to examine potential alterations in depression-like states in rats. The immobility time of the rats during the 300 s test served as an indicator of depressive behavior. Our findings revealed that rats in the medium-dose arsenic-exposed group exhibited a significantly higher immobility time (208.93 ± 16.07 s) compared to the control group (56.26 ± 20.92 s) (*p* < 0.001, Figure 6B). These results indicate that arsenic exposure at doses exceeding 10 mg/L may induce depressive-like states in rats.

### 3.3. Evaluated for Arsenic Exposure

**Figure 7:**
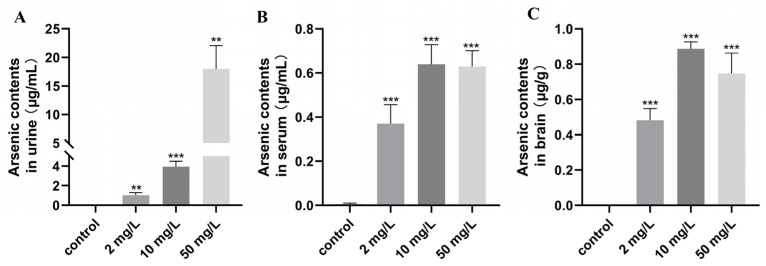
Arsenic contents. (**A**) Arsenic contents in urine. (**B**) Arsenic contents in serum. (**C**) Arsenic contents in the brain. Values are the mean ± SD. ** *p* < 0.01, *** *p* < 0.001, compared with control group, *n* = 6.

After the behavioral experiments, the rats were evaluated for arsenic exposure. The results revealed significant differences in arsenic levels in urine, serum, and brain tissue between the control group and the various arsenic-exposed groups. The urinary arsenic levels showed a dose-dependent relationship, with the highest levels observed in the high-dose arsenic-exposed group, reaching 18.00 ± 4.05 μg/mL (Figure 7A). In terms of serum arsenic levels, both the medium-dose and high-dose arsenic-exposed groups exhibited similar levels, with values of 0.64 ± 0.09 μg/mL and 0.63 ± 0.07 μg/mL, respectively (Figure 7B). The medium-dose arsenic-exposed group had the highest content of arsenic in the brain tissue, measuring 0.87 ± 0.04 μg/g (Figure 7C). These findings indicate that arsenic exposure resulted in increased urinary, serum, and brain tissue arsenic levels.

### 3.4. Arsenic Exposure Induces Hippocampal Damage Revealed by HE Staining

**Figure 8:**
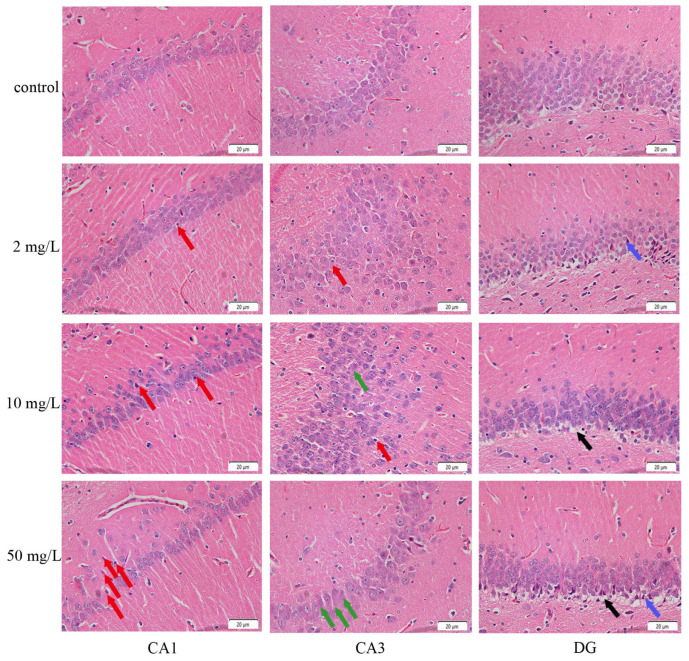
Hematoxylin–eosin staining of the hippocampus after continuous arsenic exposure in rats (×400). Red arrow: nucleus pyknosis. Green arrow: unclear cell membrane boundary. Black arrow: cell vacuolation. Blue arrow: red neuron.

HE staining revealed varying degrees of damage in the CA1, CA3, and DG regions of the hippocampus in the arsenic-exposed groups. In contrast, the control group exhibited uniformly sized neuronal cells that were closely packed together, with nuclei displaying distinct nuclear membrane borders and visible nucleoli. In the CA1 region, the staining of low-, medium-, and high-dose arsenic-exposed groups showed different levels of nuclear shrinkage, indicated by red arrows. The high-dose arsenic-exposed group exhibited a significant reduction in the number of cells (Figure 8). In the CA3 region, the low- and medium-dose arsenic-exposed groups displayed noticeable alterations, including cell proliferation, irregular structure, and disordered arrangement. The high-dose arsenic-exposed group showed an unclear nuclear membrane boundary and absence of nucleoli, as indicated by green arrows (Figure 8). In the DG region, the appearance of red neurons was observed in the low-dose and the high-dose arsenic-exposed group, as shown by blue arrows. The degree of vacuolization of the underlying cells was enhanced with the increase in arsenic exposure, as shown by black arrows (Figure 8). These findings suggest that continuous arsenic exposure can induce significant damage to the hippocampus, with specific effects observed in different regions of this brain structure.

## 4. Discussion

This study aimed to establish a subchronic model of arsenic exposure through drinking water, to investigate its effects on nervous system damage in rats. Unlike previous studies that primarily focused on memory and cognitive function, this study conducted a comprehensive assessment of neurobehavioral function. It examined various domains, including short-term memory, spatial learning and memory, anxiety-like behavior, and depression-like states. By analyzing a wide range of behavioral parameters, the study aimed to obtain a holistic understanding of the neurological impact of arsenic exposure. Furthermore, histopathological evidence strengthens the understanding of the underlying mechanisms and provides a direct link between arsenic exposure and hippocampal damage.

In the open field experiment, the residence time and movement distance in the central area of the rats served as indicators of their curiosity towards the new environment [27]. Although the exploratory abilities of the arsenic-exposed rats in this study showed a decrease, the difference compared to the control group was not statistically significant. Taheri et al. conducted a similar open field experiment on adult Wistar rats exposed to arsenic, revealing a reduced total distance and movement speed in the arsenic-exposed rats; however, their study did not mention the residence time and movement distance in the central region [12]. Previous investigations have reported significant reductions in both movement distance and residence time in the central region, for C57BL/6 male mice exposed to arsenic trioxide and female offspring mice exposed to sodium arsenite [27,28]. However, there are discrepancies in terms of species and arsenic exposure doses between the aforementioned studies and the current investigation. Specifically, this study employed lower doses of arsenic and a longer duration of exposure. To enhance the accuracy and reliability of the results, it is recommended to increase the sample size for further validation.

The Y maze and Morris water maze tests are widely regarded as gold-standard behavioral methods for assessing memory abilities in laboratory animals. The Y maze test evaluates spontaneous alternation behavior, which reflects short-term memory, while the Morris water maze assesses spatial learning and memory by measuring the ability of animals to locate a hidden platform in a pool [22]. In this study, the results revealed that arsenic exposure at concentrations above 10 mg/L had a significant detrimental impact on both short-term memory and spatial memory abilities. Previous studies have also reported a decline in spatial memory abilities in rats exposed to sodium arsenite concentrations exceeding 5 mg/L in drinking water over a 6-month period [19]. Although there are variations in exposure duration and dosage compared to our study, the consistent findings indicate a robust association between arsenic exposure and spatial memory impairment.

Currently, research on arsenic-induced anxiety primarily focuses on the developmental toxicity during pregnancy in rats. Studies have shown that even low levels of arsenic exposure in newborn rats can result in decreased social skills and increased anxiety-like behavior [13]. Additionally, Lu et al. discovered that exposure to arsenic during pregnancy induces anxiety-like behavior in adult mice [29]. In this study, we employed the elevated cross maze experiment to evaluate anxiety levels in rats exposed to arsenic. The findings indicate that postnatal exposure to arsenic, initiated at four weeks of age and lasting for three months, resulted in the development of anxiety-like behaviors in the rats. This highlights the potential long-term consequences of arsenic exposure during early life stages on anxiety-related behaviors.

Limited animal studies have been conducted to explore the association between arsenic exposure and depression. A study revealed that exposing normal mice to 10 mg/L of arsenic trioxide in drinking water for 4 weeks induced anxiety-like behavior but did not lead to a depressive state. However, in mouse models of depression induced by reserpine, it took 8 weeks of subchronic arsenic exposure to enhance depression-like behavior [30]. In another study, Oreen Samad et al. administered intraperitoneal injections of 2.5 mg/kg/mL of inorganic arsenic to rats, and after 4 weeks of exposure, the rats exhibited symptoms of depression [31]. In our study, we aimed to replicate the common pathway of arsenic exposure observed in the general population, by using drinking water as the exposure method. We found that rats exposed to drinking water containing 10 mg/L of sodium arsenite for three months exhibited a significant incidence of depressive symptoms. Although we did not observe a statistically significant difference in immobility time between the high-dose arsenic-exposed group and the control group, the presence of large inter-individual differences within the 50 mg/L group might have influenced this outcome. Considering that our experiment cannot be replicated within a short timeframe and there are currently no comparable studies available, it is necessary to conduct further research to validate our findings. By expanding the scope to include these psychological outcomes, the study sheds light on the broader behavioral consequences of arsenic exposure and highlights the importance of considering mental health effects in arsenic toxicity research.

Several studies have shed light on the concerning association between arsenic exposure and detrimental psychological effects. A survey conducted on Italian schoolchildren found that urinary arsenic levels were linked to the emergence of depression, anxiety, somatic problems, and attention disorders [32]. In a US population study, it was discovered that elevated urinary arsenate acid levels may increase the risk of depression [33]. Furthermore, Dona Sinha’s research demonstrated a correlation between the incidence of neurobehavioral symptoms, depression, and chronic low-level arsenic exposure among Indian women of childbearing age [34]. These findings highlight, from a population perspective, that arsenic exposure has been associated with impaired intellectual development, depression, anxiety, attention disorders, and neurobehavioral symptoms.

In this study, male rats were chosen as the subjects to investigate the effects of poisons. This selection was primarily based on the fact that male rats exhibit greater stability in their response to poisons and are less susceptible to hormonal influences compared to their female counterparts [35,36]. Furthermore, male rats demonstrate a relatively consistent pattern of physiological and social behavior [37], thereby minimizing confounding factors and enhancing the reliability and comparability of the study results. Arsenic, typically ingested as arsenite, is found in the environment [38]. Previous studies utilized sodium arsenite solutions of various concentrations (0, 5, 10, and 50 mg/L) for 3 months to induce arsenic poisoning in rats [19,20]. This study considered the exposure duration and doses used in previous research. However, these doses exceed human exposure under natural conditions. For instance, in regions like Bangladesh with high levels of arsenic in drinking water, the maximum exposure dose is 4.73 mg/L [39]. Considering the equivalent dose conversion based on body surface area, 4.73 mg/L in humans is approximately equivalent to 27.91 mg/L in rats, falling between the highest (50 mg/L) and medium (10 mg/L) doses used in this study. Thus, the rat exposure doses provide valuable insights into human exposure conditions. Nevertheless, it is important to acknowledge the uncertainty in extrapolating rodent results to humans, due to the differences in their sensitivity to toxic chemicals. Future studies will investigate additional arsenic exposure doses and time points to enhance our understanding of arsenic’s neurotoxic effects.

The toxic effects of arsenic are directly associated with the level of exposure. Although water intake may have decreased in the arsenic-exposed groups, potentially due to taste aversion or physiological reactions to sodium arsenite, the actual intake of arsenic reflected an increase in total arsenic intake with higher exposure concentrations. Assessing the concentration of arsenic in biological samples can serve as an indicator of the body’s arsenic burden. Urinary arsenic levels generally reflect short-term exposure, while serum arsenic and brain arsenic levels are indicative of accumulated levels over time. In our study, we observed a positive correlation between arsenic concentration and the levels of urinary, serum, and brain arsenic in rats. As the arsenic concentration increased, so did the levels of arsenic detected in these samples. Interestingly, we found that the medium-dose arsenic-exposed group exhibited comparable serum and brain arsenic levels to the high-dose arsenic-exposed group. Prior research has demonstrated that higher levels of arsenic concentration are associated with increased brain arsenic levels. However, when the arsenic concentration rises exponentially, the corresponding changes in brain arsenic levels do not exhibit the same exponential pattern [40]. Combined with the results of this study, it can be shown that the brain has a limited capacity to absorb and accumulate arsenic, possibly due to protective mechanisms or processes that regulate the uptake and distribution of arsenic in the brain.

These findings align with the results of the behavioral experiments. The accumulation of arsenic in the brain tissues of rats in the medium-dose arsenic-exposed group and the high-dose arsenic-exposed group exhibited similarities, resulting in similar behavioral effects. In both the Y maze experiment and the forced swimming experiment, the rats in the high-dose arsenic-exposed group performed slightly better than those exposed to medium doses. This discrepancy in behavior may be attributed to the unique accumulation pattern of arsenic in brain tissue.

The exact mechanism underlying the neurotoxic effects of arsenic is not yet fully understood. However, current research suggests that the toxic effects of arsenic primarily stem from various pathological factors, including oxidative stress [41], inflammation [42], apoptosis [19], and synaptic plasticity [43]. Studies have indicated that arsenic can contribute to nerve damage by inducing an inflammatory response in the hippocampus [44]. Additionally, arsenic has been found to impact hippocampal autophagy [45], mitochondrial function [46], and synaptic function [47]. As a result, this particular study aimed to investigate the pathological damage inflicted on hippocampal tissue due to arsenic exposure.

The hippocampus, a crucial brain region implicated in various neural functions, was the focus of this study, in which comprehensive pathological observations across the hippocampus’ different regions in rats were conducted. The results revealed varying degrees of pathological damage in the CA1, CA3, and DG regions of the hippocampus in subchronic arsenic-exposed rats. Arsenic-exposed rats exhibited a reduced pyramidal cell count in the CA1 region, resembling the neuropathological alterations seen in sevoflurane-induced nerve injury [48]. Furthermore, treatment with reserpine induced depression-like symptoms and disrupted pyramidal cell arrangement in the CA1 region. These alterations in nerve cell morphology within the CA1 region have implications for signal processing and synaptic function, which are closely associated with long-term memory, including conditions like Alzheimer’s disease [49]. Similarly, the CA3 region of the hippocampus exhibited evident cell proliferation and disordered arrangement in response to arsenic exposure, consistent with previous findings by Ping Tian et al. [50,51].

In the DG region, vacuolation was observed in the underlying neurons in response to arsenic exposure. The vacuolization of red neurons and underlying cells were observed in the DG region of rats exposed to arsenic. “Red neurons” refers to the term “acute necrosis of neurons,” which describes a morphological change that occurs due to ischemia, hypoxia, infection, or poisoning. Studies have demonstrated that neuronal vacuolation is linked to neuronal death [52]. Based on the HE staining results, we speculate that contamination of toxic substances in the hippocampal DG area initially leads to acute necrosis of neurons. Subsequently, there is a progressive escalation of neuronal damage, eventually resulting in neuronal vacuolization.

The hippocampal DG–CA3 circuit, responsible for memory encoding and retrieval, is highly susceptible to stress. Under conditions of early life stress, the loss of GABAergic NPY+ cells may lead to reduced inhibition within the DG–CA3 pathway, resulting in hyperexcitability and subsequent deficits in hippocampal-dependent behaviors [53]. As the “gateway” regulating information flows into the hippocampus, the DG plays a critical role in cognitive and emotional processes [54]. Exposure to morphine in adult offspring induced anxiety-like behavior and dendritic retractions specifically in the DG region of the hippocampus [55]. Additionally, studies have shown that anxiety-like behavior in rats is associated with decreased neuronal differentiation in the DG [56]. These findings shed light on the pathological effects of arsenic exposure on the hippocampus and its potential behavioral consequences. The primary objective of this study was to examine the behavioral changes and pathological damage in the hippocampus of rats exposed to arsenic. However, regrettably, this paper does not delve into the precise mechanisms and molecular pathways underlying these effects. By delving deeper into the mechanistic aspects, future studies can provide a more comprehensive understanding of how arsenic affects the hippocampus at the molecular level.

## 5. Conclusions

In summary, the study demonstrated that subchronic drinking water exposure to arsenic led to a decline in neurobehavioral function and pathological damage in various regions of the hippocampus in rats. The findings underscore the association between arsenic exposure and impairments in memory, anxiety-like behavior, and depression-like symptoms, providing valuable insights into the harmful effects of arsenic on the hippocampus and its repercussions on cognitive and emotional functions.

## Data Availability

Data are contained within the article.

## References

[1] WHO Guidelines Approved by the Guidelines Review Committee[A] (2017). Guidelines for Drinking-Water Quality: Fourth Edition Incorporating the First Addendum.

[2] Podgorski J., Berg M. (2020). Global threat of arsenic in groundwater. Science.

[3] Raessler M. (2018). The Arsenic Contamination of Drinking and Groundwaters in Bangladesh: Featuring Biogeochemical Aspects and Implications on Public Health. Arch. Environ. Contam. Toxicol..

[4] Iyer S., Sengupta C., Velumani A. (2016). Blood arsenic: Pan-India prevalence. Clin. Chim. Acta.

[5] Han L., Gao B., Hao H., Lu J., Xu D. (2019). Arsenic pollution of sediments in China: An assessment by geochemical baseline. Sci. Total Environ..

[6] Tapia J., Murray J., Ormachea M., Tirado N., Nordstrom D.K. (2019). Origin, distribution, and geochemistry of arsenic in the Altiplano-Puna plateau of Argentina, Bolivia, Chile, and Perú. Sci. Total Environ..

[7] Mañay N., Pistón M., Cáceres M., Pizzorno P., Bühl V. (2019). An overview of environmental arsenic issues and exposure risks in Uruguay. Sci. Total Environ..

[8] Raju N.J. (2022). Arsenic in the geo-environment: A review of sources, geochemical processes, toxicity and removal technologies. Environ. Res..

[9] Wasserman G.A., Liu X., Parvez F., Chen Y., Factor-Litvak P., LoIacono N.J., Levy D., Shahriar H., Uddin M.N., Islam T. (2018). A cross-sectional study of water arsenic exposure and intellectual function in adolescence in Araihazar, Bangladesh. Environ. Int..

[10] Hou Y., Zhao W., Yu H., Zhang F., Zhang H.T., Zhou Y. (2022). Biochanin A alleviates cognitive impairment and hippocampal mitochondrial damage in ovariectomized APP/PS1 mice. Phytomedicine.

[11] Kumar Arora M., Ratra A., Asdaq S.M.B., Alshamrani A.A., Alsalman A.J., Kamal M., Tomar R., Sahoo J., Ashok J., Imran M. (2022). Plumbagin Alleviates Intracerebroventricular-Quinolinic Acid Induced Depression-like Behavior and Memory Deficits in Wistar Rats. Molecules.

[12] Taheri Zadeh Z., Esmaeilpour K., Aminzadeh A., Heidari M.R., Joushi S. (2021). Resveratrol Attenuates Learning, Memory, and Social Interaction Impairments in Rats Exposed to Arsenic. BioMed Res. Int..

[13] Zhou H., Zhao W., Ye L., Chen Z., Cui Y. (2018). Postnatal low-concentration arsenic exposure induces autism-like behavior and affects frontal cortex neurogenesis in rats. Environ. Toxicol. Pharmacol..

[14] Karri V., Ramos D., Martinez J.B., Odena A., Oliveira E., Coort S.L., Evelo C.T., Mariman E.C.M., Schuhmacher M., Kumar V. (2018). Differential protein expression of hippocampal cells associated with heavy metals (Pb, As, and MeHg) neurotoxicity: Deepening into the molecular mechanism of neurodegenerative diseases. J. Proteom..

[15] Lathe R., Singadia S., Jordan C., Riedel G. (2020). The interoceptive hippocampus: Mouse brain endocrine receptor expression highlights a dentate gyrus (DG)-cornu ammonis (CA) challenge-sufficiency axis. PLoS ONE.

[16] Liang X., Hsu L.M., Lu H., Ash J.A., Rapp P.R., Yang Y. (2020). Functional Connectivity of Hippocampal CA3 Predicts Neurocognitive Aging via CA1-Frontal Circuit. Cereb. Cortex.

[17] Cheng J., Scala F., Blanco F.A., Niu S., Firozi K., Keehan L., Mulherkar S., Froudarakis E., Li L., Duman J.G. (2021). The Rac-GEF Tiam1 Promotes Dendrite and Synapse Stabilization of Dentate Granule Cells and Restricts Hippocampal-Dependent Memory Functions. J. Neurosci..

[18] Qu L., Gao Y., Sun H., Wang H., Liu X., Sun D. (2016). Role of PTEN-Akt-CREB Signaling Pathway in Nervous System impairment of Rats with Chronic Arsenite Exposure. Biol. Trace Elem. Res..

[19] Sun H., Yang Y., Shao H., Sun W., Gu M., Wang H., Jiang L., Qu L., Sun D., Gao Y. (2017). Sodium Arsenite-Induced Learning and Memory Impairment Is Associated with Endoplasmic Reticulum Stress-Mediated Apoptosis in Rat Hippocampus. Front. Mol. Neurosci..

[20] Sun H., Yang Y., Gu M., Li Y., Jiao Z., Lu C., Li B., Jiang Y., Jiang L., Chu F. (2022). The role of Fas-FasL-FADD signaling pathway in arsenic-mediated neuronal apoptosis in vivo and in vitro. Toxicol. Lett..

[21] Wang J.Y., Zhang Y., Chen Y., Wang Y., Li S.Y., Wang Y.F., Zhang Z.X., Zhang J., Rong P. (2021). Mechanisms underlying antidepressant effect of transcutaneous auricular vagus nerve stimulation on CUMS model rats based on hippocampal α7nAchR/NF-κB signal pathway. J. Neuroinflamm..

[22] Kraeuter A.K., Guest P.C., Sarnyai Z. (2019). The Y-Maze for Assessment of Spatial Working and Reference Memory in Mice. Methods Mol. Biol..

[23] Hu C., Luo Y., Wang H., Kuang S., Liang G., Yang Y., Mai S., Yang J. (2017). Re-evaluation of the interrelationships among the behavioral tests in rats exposed to chronic unpredictable mild stress. PLoS ONE.

[24] Yankelevitch-Yahav R., Franko M., Huly A., Doron R. (2015). The forced swim test as a model of depressive-like behavior. J. Vis. Exp..

[25] Xie W.J., Che L., Zhou G.Y., Yang L.N., Hu M.Y. (2016). The bioconcentration ability of heavy metal research for 50 kinds of rice under the same test conditions. Environ. Monit. Assess..

[26] Ding B., Ma X., Liu Y., Ni B., Lu S., Chen Y., Liu X., Zhang W. (2023). Arsenic-Induced, Mitochondria-Mediated Apoptosis Is Associated with Decreased Peroxisome Proliferator-Activated Receptor γ Coactivator α in Rat Brains. Toxics.

[27] Hu X., Yuan X., Yang M., Han M., Ommati M.M., Ma Y. (2023). Arsenic exposure induced anxiety-like behaviors in male mice via influencing the GABAergic Signaling in the prefrontal cortex. Environ. Sci. Pollut. Res. Int..

[28] Kyi-Tha-Thu C., Htway S.M., Suzuki T., Nohara K., Win-Shwe T.T. (2023). Gestational arsenic exposure induces anxiety-like behaviors in F1 female mice by dysregulation of neurological and immunological markers. Environ. Health Prev. Med..

[29] Lv J.W., Song Y.P., Zhang Z.C., Fan Y.J., Xu F.X., Gao L., Zhang X.Y., Zhang C., Wang H., Xu D.X. (2021). Gestational arsenic exposure induces anxiety-like behaviors in adult offspring by reducing DNA hydroxymethylation in the developing brain. Ecotoxicol. Environ. Saf..

[30] Chang C.Y., Guo H.R., Tsai W.C., Yang K.L., Lin L.C., Cheng T.J., Chuu J.J. (2015). Subchronic Arsenic Exposure Induces Anxiety-Like Behaviors in Normal Mice and Enhances Depression-Like Behaviors in the Chemically Induced Mouse Model of Depression. BioMed Res. Int..

[31] Samad N., Jabeen S., Imran I., Zulfiqar I., Bilal K. (2019). Protective effect of gallic acid against arsenic-induced anxiety-/depression- like behaviors and memory impairment in male rats. Metab. Brain Dis..

[32] Renzetti S., Cagna G., Calza S., Conversano M., Fedrighi C., Forte G., Giorgino A., Guazzetti S., Majorani C., Oppini M. (2021). The effects of the exposure to neurotoxic elements on Italian schoolchildren behavior. Sci. Rep..

[33] Rahman H.H., Yusuf K.K., Niemann D., Dipon S.R. (2020). Urinary speciated arsenic and depression among US adults. Environ. Sci. Pollut. Res. Int..

[34] Mukherjee B., Bindhani B., Saha H., Sinha D., Ray M.R. (2014). Platelet hyperactivity, neurobehavioral symptoms and depression among Indian women chronically exposed to low level of arsenic. Neurotoxicology.

[35] Renczés E., Borbélyová V., Steinhardt M., Höpfner T., Stehle T., Ostatníková D., Celec P. (2020). The Role of Estrogen in Anxiety-Like Behavior and Memory of Middle-Aged Female Rats. Front. Endocrinol. (Lausanne).

[36] Hawley W.R., Grissom E.M., Barratt H.E., Conrad T.S., Dohanich G.P. (2012). The effects of biological sex and gonadal hormones on learning strategy in adult rats. Physiol. Behav..

[37] Vanderschuren L.J., Trezza V. (2014). What the laboratory rat has taught us about social play behavior: Role in behavioral development and neural mechanisms. Curr. Top. Behav. Neurosci..

[38] Kaur S., Kamli M.R., Ali A. (2011). Role of arsenic and its resistance in nature. Can. J. Microbiol..

[39] Chakraborti D., Rahman M.M., Mukherjee A., Alauddin M., Hassan M., Dutta R.N., Pati S., Mukherjee S.C., Roy S., Quamruzzman Q. (2015). Groundwater arsenic contamination in Bangladesh-21 Years of research. J. Trace Elem. Med. Biol..

[40] Yi Y., Gao S., Xia J., Li C., Zhao Y., Zhang Y., Liang A., Ji S. (2019). Data on the sub-chronic toxicity in rats after 30 days of oral realgar administration and the accumulation and distribution of arsenic species. Data Brief..

[41] González-Alfonso W.L., Pavel P., Karina H.M., Del Razo L.M., Sanchez-Peña L.C., Zepeda A., Gonsebatt M.E. (2023). Chronic exposure to inorganic arsenic and fluoride induces redox imbalance, inhibits the transsulfuration pathway, and alters glutamate receptor expression in the brain, resulting in memory impairment in adult male mouse offspring. Arch. Toxicol..

[42] Liu X., Zhang R., Fan J., Chen Y., Wang H., Ge Y., Liang H., Li W., Liu H., Lv Z. (2023). The role of ROS/p38 MAPK/NLRP3 inflammasome cascade in arsenic-induced depression-/anxiety-like behaviors of mice. Ecotoxicol. Environ. Saf..

[43] Liu X., Wang J. (2022). N-Methyl-D-Aspartate Receptors Mediate Synaptic Plasticity Impairment of Hippocampal Neurons Due to Arsenic Exposure. Neuroscience.

[44] Jing H., Yan N., Fan R., Li Z., Wang Q., Xu K., Hu X., Zhang L., Duan X. (2023). Arsenic Activates the NLRP3 Inflammasome and Disturbs the Th1/Th2/Th17/Treg Balance in the Hippocampus in Mice. Biol. Trace Elem. Res..

[45] Manthari R.K., Tikka C., Ommati M.M., Niu R., Sun Z., Wang J., Zhang J., Wang J. (2018). Arsenic induces autophagy in developmental mouse cerebral cortex and hippocampus by inhibiting PI3K/Akt/mTOR signaling pathway: Involvement of blood-brain barrier’s tight junction proteins. Arch. Toxicol..

[46] Keshavarz-Bahaghighat H., Sepand M.R., Ghahremani M.H., Aghsami M., Sanadgol N., Omidi A., Bodaghi-Namileh V., Sabzevari O. (2018). Acetyl-L-Carnitine Attenuates Arsenic-Induced Oxidative Stress and Hippocampal Mitochondrial Dysfunction. Biol. Trace Elem Res..

[47] Foley K.F.W., Barnett D., Cory-Slechta D.A., Xia H. (2021). Early Low-Level Arsenic Exposure Impacts Post-Synaptic Hippocampal Function in Juvenile Mice. Toxics.

[48] Chen Y., Gao X., Pei H. (2022). miRNA-384-3p alleviates sevoflurane-induced nerve injury by inhibiting Aak1 kinase in neonatal rats. Brain Behav..

[49] Olanrewaju J.A., Owolabi J.O., Awodein I.P., Enya J.I., Adelodun S.T., Olatunji S.Y., Fabiyi S.O. (2020). Zingiber officinale Ethanolic Extract Attenuated Reserpine-Induced Depression-Like Condition and Associated Hippocampal Aberrations in Experimental Wistar Rats. J. Exp. Pharmacol..

[50] Tian P., Zhang W., Li K.Y., Li H.W., Ma K., Han D.E. (2022). Effect of Rehmanniae Radix on depression-like behavior and hippocampal monoamine neurotransmitters of chronic unpredictable mild stress model rats. Zhongguo Zhong Yao Za Zhi.

[51] Konakanchi S., Raavi V., Ml H.K., Shankar Ms V. (2022). Effect of chronic sleep deprivation and sleep recovery on hippocampal CA3 neurons, spatial memory and anxiety-like behavior in rats. Neurobiol. Learn. Mem..

[52] Krinke G.J. (2011). Neuronal vacuolation. Toxicol. Pathol..

[53] Alviña K., Jodeiri Farshbaf M., Mondal A.K. (2021). Long term effects of stress on hippocampal function: Emphasis on early life stress paradigms and potential involvement of neuropeptide Y. J. Neurosci. Res..

[54] Sun D., Milibari L., Pan J.X., Ren X., Yao L.L., Zhao Y., Shen C., Chen W.B., Tang F.L., Lee D. (2021). Critical Roles of Embryonic Born Dorsal Dentate Granule Neurons for Activity-Dependent Increases in BDNF, Adult Hippocampal Neurogenesis, and Antianxiety-like Behaviors. Biol. Psychiatry.

[55] Li C.Q., Luo Y.W., Bi F.F., Cui T.T., Song L., Cao W.Y., Zhang J.Y., Li F., Xu J.M., Hao W. (2014). Development of anxiety-like behavior via hippocampal IGF-2 signaling in the offspring of parental morphine exposure: Effect of enriched environment. Neuropsychopharmacology.

[56] Du Preez A., Law T., Onorato D., Lim Y.M., Eiben P., Musaelyan K., Egeland M., Hye A., Zunszain P.A., Thuret S. (2020). The type of stress matters: Repeated injection and permanent social isolation stress in male mice have a differential effect on anxiety- and depressive-like behaviours, and associated biological alterations. Transl. Psychiatry.

